# The *Pseudomonas syringae* pv. tomato DC3000 PSPTO_0820 multidrug transporter is involved in resistance to plant antimicrobials and bacterial survival during tomato plant infection

**DOI:** 10.1371/journal.pone.0218815

**Published:** 2019-06-25

**Authors:** Saray Santamaría-Hernando, Marta Senovilla, Almudena González-Mula, Pedro Manuel Martínez-García, Sandra Nebreda, Pablo Rodríguez-Palenzuela, Emilia López-Solanilla, José Juan Rodríguez-Herva

**Affiliations:** 1 Centro de Biotecnología y Genómica de Plantas (CBGP), Universidad Politécnica de Madrid-Instituto Nacional de Investigación y Tecnología Agraria y Alimentaria, Pozuelo de Alarcón, Madrid, Spain; 2 Departamento de Biotecnología-Biología Vegetal, Escuela Técnica Superior de Ingeniería Agronómica, Alimentaria y de Biosistemas, Universidad Politécnica de Madrid, Madrid, Spain; ICAR-Indian Institute of Agricultural Biotechnology, INDIA

## Abstract

Multidrug resistance efflux pumps protect bacterial cells against a wide spectrum of antimicrobial compounds. PSPTO_0820 is a predicted multidrug transporter from the phytopathogenic bacterium *Pseudomonas syringae* pv. tomato DC3000. Orthologs of this protein are conserved within many *Pseudomonas* species that interact with plants. To study the potential role of *PSPTO_0820* in plant-bacteria interaction, a mutant in this gene was isolated and characterized. In addition, with the aim to find the outer membrane channel for this efflux system, a mutant in *PSPTO_4977*, a TolC-like gene, was also analyzed. Both mutants were more susceptible to *trans*-cinnamic and chlorogenic acids and to the flavonoid (+)-catechin, when added to the culture medium. The expression level of both genes increased in the presence of (+)-catechin and, in the case of *PSPTO_0820*, also in response to *trans*-cinnamic acid. PSPTO_0820 and PSPTO_4977 mutants were unable to colonize tomato at high population levels. This work evidences the involvement of these two proteins in the resistance to plant antimicrobials, supporting also the importance of chlorogenic acid, *trans*-cinnamic acid, and (+)-catechin in the tomato plant defense response against *P*. *syringae* pv. tomato DC3000 infection.

## Introduction

*Pseudomonas syringae* pv. tomato DC3000 (*PsPto*) is a phytopatogenic bacterium that infects tomato (causing bacterial speck) and *Arabidopsis thaliana*. *PsPto* can grow epiphytically and endophytically on plant foliage without causing disease symptoms [1]. In the early stages of the infective phase, *PsPto* enters the plant through wounds and natural openings (such as stomata) and multiplies in the apoplastic space by exploiting live host cells. In this scenario, bacterial survival in the apoplast is one of the key factors for the establishment of a bacterial density large enough to further infect adjacent plant tissues [2]. However, plant apoplast represents a harsh environment for bacteria since it is laden with antimicrobial compounds, both preformed (phytoanticipins) and inducible (phytoalexins), which constitute chemical barriers capable of inhibiting the growth of the pathogen. In fact, plants produce antimicrobial peptides and a variety of secondary metabolites such as phenylpropanoids, isoprenoids, and alkaloids, that are generally accepted to play a role in protecting plants against pathogens. Mode of action of these compounds has been elucidated in some cases [3–6]. Using the tomato-*PsPto* pathosystem, an increased expression of phenylpropanoid biosynthetic genes was detected upon bacterial infection, with specific accumulation of different phenylpropanoids such as hydroxycinnamic acid amides conjugated to alkaloids, chlorogenic acid (CGA), and the flavonoid rutin [7–9]. Tomato plants have also been reported to produce other number of flavonoids like chalconaringenin, rutin, quercetin 3-*O*-(2"-*O*-ß-apiosyl-6"-*O*-α-rhamnosyl-ß-glucoside) or phloretin 3', 5'-di-*C*-ß-glucoside [10, 11]. To overcome the effect of these potentially toxic compounds, plant-associated bacteria have in turn evolved different defense strategies, among which multidrug resistance (MDR) efflux pumps are the most widespread. MDR transporters can recognize and pump out many different organic compounds (often structurally dissimilar), providing resistance to antibiotics and many other antimicrobial compounds [12, 13]. Microorganisms with the largest number of MDR pumps are found in the soil or in association with plants [14, 15]. Although still scarce, several studies on plant-pathogen interactions with bacteria from the genera *Xanthomonas*, *Ralstonia*, *Erwinia* and *Dickeya* have shown that efflux pumps can contribute to bacterial virulence, bacterial fitness, resistance to plant antimicrobials, or competition with epiphytic bacteria [16–21].

Regarding *P*. *syringae*, most studies have been focused on MexAB-OprM, an efflux pump from the resistance-nodulation-cell division (RND) family. RND efflux systems exist in a tripartite form, consisting of an inner membrane transporter which recognizes and transports the substrates, a periplasmic adaptor protein belonging to the membrane fusion protein (MFP) family and an outer membrane channel belonging to outer membrane factor (OMF) family [22, 23]. Stoitsova et al. [24] showed that the *P*. *syringae* MexAB-OprM system is involved in the tolerance to a broad range of toxic compounds, including some plant-derived antimicrobials, and that a mutant in this system showed a reduced ability to multiply *in planta*. Vargas et al. [25] carried out a detailed analysis on the regulation of the expression of this MDR, which is controlled by the PmeR repressor and specifically induced by flavonoids. These authors also found that different tomato flavonoids, such as morin, naringenin and phloretin, reduce the expression of the *PsPto* type III secretion system and also inhibit swimming and swarming motility in this bacterium by decreasing the expression of flagella [26]. A recent study on the Arabidopsis-*PsPto* pathosystem has identified three RND efflux pumps (one of them the MexAB-OprM system) which are required to overcome the isothiocyanate-based defenses of Arabidopsis [27].

With the aim to find out new, still not characterized, *PsPto* MDR transporters from the RND family involved in plant-bacteria interaction, we focused our attention in PSPTO_0820, which has orthologs in many plant-pathogenic and plant-associated species within the *Pseudomonas* genus. In addition, we also studied the role of PSPTO_4977, a TolC-like outer membrane efflux protein potentially related to the previous one. To determine whether these proteins contributed to *PsPto* plant colonization ability, mutants in these two genes were isolated, tested for their resistance against plant-derived antimicrobials, and checked for their phenotype in plant infection assays. We found that these proteins are involved in bacterial resistance to various plant antimicrobials, such as chlorogenic and *trans*-cinnamic acids and (+)-catechin, and that they have a relevant role in the survival of *PsPto* in tomato plants.

## Materials and methods

### Bacterial strains and growth conditions

Bacterial strains used in this work are listed in Table 1. *Escherichia coli* strains were routinely grown in liquid Luria-Bertani (LB) medium at 37°C [28]. *PsPto* strains were grown in King’s B (KB) medium at 28°C [29]. Nutrient broth (N1) medium [30] was used in some of the microbial susceptibility assays. When required, antibiotics were added to media at the following final concentrations (μg/ml): ampicillin, 100; gentamycin, 1 (for *Pseudomonas*) or 5 (for *E*. *coli*); kanamycin, 30; nitrofurantoin, 5; rifampicin, 25; and streptomycin, 50.

**Table 1:** Bacterial strains and plasmids used in this study.

Strain or plasmid	Relevant characteristics ^*a*^	Reference or source
Strains		
*E*. *coli*		
DG1	*mcrA* Δ(*mrr-hsdRMS-mcrBC*) ϕ80*lacZ*ΔM15 Δ*lac*X74 *recA*1 *araD*139 Δ(*ara-leu*)7697 *galU galK rpsL endA*1 *nupG*	Eurogentec
*P*. *syringae* pv. tomato DC3000		
*PsPto*	Wild-type; Rif^r^	[31]
PS0820	DC3000 *PSPTO_0820*::mini-Tn*5gusA*Gm (insertion between positions 1467 and 1468 of the coding sequence); Gm^r^	This study
PS4977	DC3000 *PSPTO_4977*::mini-Tn*5gusA*Gm (insertion between positions 277 and 278 of the coding sequence); Gm^r^	This study
PS0820 (pBBR0820)	PS0820 mutant harboring the pBBR0820 plasmid; Gm^r^, Km^r^	This study
PS4977 (pBBR4977)	PS4977 mutant harboring the pBBR4977 plasmid; Gm^r^, Km^r^	This study
Plasmids		
pBBR1MCS-2	Km^r^; broad-host range, *oriT*RK2, α-*lacZ*	[32]
pBBR1MCS-5	Gm^r^; broad-host range, *oriT*RK2, α-*lacZ*	[32]
pHP45Ω	Ap^r^, Sm^r^/Sp^r^; *ori*ColE1, Ω- Sm^r^/Sp^r^ interposon	[33]
pUC18Sfi	Ap^r^; identical to pUC18 but with SfiI sites flanking the polylinker	[34]
pCAM140	Sm^r^/Sp^r^, Ap^r^, *ori*R6K, *ori*TRK2; pUT/mini-Tn*5*SS*gusA*40 carrying a Ω–Sm^r^/Sp^r^ interposon and promoterless *gusA* gene	[35]
pHP45ΩGm	Gm^r^; pHP45Ω carrying a Gm^r^ cassette (as a SphI-BssHII PCR fragment obtained from pBBR1MCS-5) replacing the original Sm^r^/Sp^r^ gene	This study
pUC18SfiGm	Ap^r^, Gm^r^; pUC18Sfi carrying, at the EcoRI site, the Ω-Gm^r^ interposon from pHP45ΩGm as an EcoRI fragment	This study
pAGM1	Gm^r^, Ap^r^, *ori*R6K, *ori*TRK2; pCAM140 carrying a mini-Tn*5gusA*Gm transposon (the SfiI Ω-Gm^r^ fragment from pUC18SfiGm replaces the original Ω-Sm^r^/Sp^r^ interposon)	This study
pBBR0820	Km^r^; pBBR1MCS-2 derivative containing an EcoRI-SacI PCR fragment carrying the *PSPTO_0820* wild-type gene (with an improved Shine-Dalgarno sequence) under the control of the P_*lac*_ promoter	This study
pBBR4977	Km^r^; pBBR1MCS-2 derivative containing a SacI PCR fragment carrying the *PSPTO_4977* wild-type gene under the control of the P_*lac*_ promoter	This study

^*a*^ Ap^r^, Gm^r^, Km^r^, Rif^r^, Sm^r^, Sp^r^, resistance to ampicillin, gentamycin, kanamycin, rifampicin, streptomycin, and spectinomycin, respectively.

### Bioinformatic analysis

Genome of *PsPto* was searched for genes encoding putative RND transporters and outer membrane efflux proteins, using a custom pipeline. To carry out the searches, different protein domains that define this class of transporters (specified by Pfam families PF02321 and PF00873) were used. The genome sequence was downloaded from the ASAP database, http://asap.ahabs.wisc.edu/asap/home.php, and protein alignments corresponding to the different Pfam domains were downloaded from the Pfam website of the Sanger Institute (http://Pfam.sanger.ac.uk/) [36]. The alignments were used to construct Hidden Markov Model (HMM) matrices. A customized BioPerl script was developed to perform HMM profile searches using HMMER v2.3.2 [37] with a cut-off E-value of 0.005. For the prediction of the presence and location of signal peptide cleavage sites in amino acid sequences, the SignalP 4.1 Server was used, http://www.cbs.dtu.dk/services/SignalP/ [38].

### Isolation of *PsPto* mutants

PS0820 and PS4977 mutants were isolated from a comprehensive transposon mutant library of *PsPto* available in our lab. Briefly, the mutant bank was generated using the pAGM1 plasmid, a pCAM140 derivative carrying a mini-Tn*5*Gm transposon with a promoterless *gusA* gene (Table 1). Mutant colonies were individually picked and grown in 96-well microtiter plates, pooled for DNA isolation, and screened by PCR, essentially as described by Holeva et al. [39]. PCR screening was performed using two different sets of primer pairs for each gene, i.e., the 5' or the 3' flanking primer plus an outward primer (GUSR2) internal to the mini-transposon (S1 Table). Inactivation of the target genes in the mutant strains was verified by PCR. Southern blot analyses were performed to confirm that the mutant strains only carried a single insertion of the transposable element in their genome (S1 Fig). Exact locations of the transposon insertions were determined by sequencing and are indicated in Table 1. Standard protocols were used for routine molecular biology methods [40].

### Construction of complemented strains

For complementation assays, the corresponding genes were PCR-amplified and cloned under the control of the P_*lac*_ promoter into the broad-host-range plasmid pBBR1MCS-2, as described in Table 1. With regard to *PSPTO_0820* gene, the amplified PCR product was provided with a canonical Shine-Dalgarno sequence (AGGAGG) to improve gene expression. Plasmids were transferred into *PsPto* mutants by electroporation. Wild-type strain carrying the pBBR1MCS-2 empty vector was used as a negative control.

### Antimicrobial susceptibility assays

Antimicrobial susceptibilities to acriflavine, carbenicillin, crystal violet, deoxycholate, novobiocin, oxacillin and rhodamine 6G (all purchased from Sigma-Aldrich) were determined by a microtiter broth dilution method. 96-well microtiter plates containing a volume of 160 μl of N1 medium per well (diluted with 1:3 of water) were inoculated with exponential-phase bacterial cells at a final concentration of 5 x 10^5^ colony forming units (CFU)/ml per well. Antimicrobials (dissolved in water) were added at different concentrations (around the minimum inhibitory concentration for DC3000) in a constant volume of 20 μl/well. Bacterial growth was determined after 24 h of incubation at 28°C with shaking (200 rpm) by measuring the culture optical density at 600 nm (OD_600_). All assays were performed in triplicate.

Minimum inhibitory concentrations (MIC) of the antibiotics ampicillin, chloramphenicol, colistin, erythromycin, sulfamethoxazole, and tetracycline were determined as described by Wang et al. [41] using Etest strips (bioMérieux), which consist of a plastic strip coated with a predefined continuous gradient of antibiotic concentrations on one side and an interpretation scale of the antimicrobial agent on the other side. KB agar (1.5% [wt/vol]) plates used in these assays were allowed to sit at room temperature overnight before use in order to achieve more consistent results. Briefly, cells were grown in liquid KB medium to an OD_600_ of 0.5. Then, 10 ml of the culture were poured on the surface of the plates and allowed to sit for 40 s. The culture was then poured out, and the excess liquid was drained. Plates were allowed to dry with their lids off for 10 min on the bench top before application of the strips, and then incubated at 28°C for 18 hours. All assays were repeated at least three times.

For assays involving plant antimicrobial compounds from the alkaloid and phenylpropanoids classes, susceptibility assays were conducted on Petri dishes (90 mm diameter) filled with 35 ml solid LB agar (0.75% [wt/vol]) as previously described [25]. Basically, exponentially growing cells (8 x 10^6^ CFU) were spread onto the medium as described above. Then, a hole (5 mm in diameter) was punched at the center of each plate using a sterile cork borer and filled with 50 μl of the different antimicrobial compounds dissolved in dimethyl sulfoxide (DMSO). After 24 h incubation at 28°C, the diameter of the growth inhibition zones in the bacterial lawn were measured. Compounds were added in the following amounts (per plate): berberin, 7.16 mg; caffeic acid, 4.5 mg; (+)-catechin, 7.25 mg; chlorogenic acid, 8.85 mg; *trans*-cinnamic acid, 3.7 mg; *p*-coumaric acid, 10.83 mg; *trans*-*p*-coumaric, 2.5 mg; esculetin, 6 mg; *trans*-ferulic acid, 4.85 mg; genistein, 0.25 mg; morin, 7.6 mg; naringenin, 9.3 mg; phloretin, 1.25 mg; phloridzin, 11.8 mg; plumbagin, 6.5 mg; quercetin, 8.5 mg; rhein, 0.5 mg; and rutin, 7.5 mg. All compounds were purchased from Sigma-Aldrich. At least five independent replicates were performed for each experiment.

### RNA extraction

Bacterial suspensions were incubated at 28°C in M9 minimal medium [28] supplemented with 1 mM MgSO_4_, 6 μg/l ammonium ferric citrate and trace metals as previously described [42] with fructose (0.5%, wt/vol) as the sole carbon source. Antimicrobial compounds were dissolved in dimethyl sulfoxide (DMSO) and added to the medium to reach a final concentration of 0.5 mM. Bacterial suspensions were incubated with shaking at 150 rpm to an OD_600_ of 0.5. Three independent biological replicates were performed for each experiment. Total RNA was extracted by using TRI Reagent solution (Invitrogen, ThermoFisher Scientific, Waltham, MA, USA) as recommended by the manufacturer. Purification was accomplished by using the High Pure RNA Isolation Kit (Roche Diagnostics, Mannheim, Germany) following the manufacturer’s recommendations. RNA samples were quantified using a NanoDrop ND1000 spectrophotometer (NanoDrop Technologies, Inc., Wilmington, DE, USA).

### Quantitative reverse transcription PCR (RT-qPCR)

Total RNA isolated as described above, was converted to cDNA using the High-Capacity cDNA Reverse Transcription Kit (Applied Biosystems, Foster City, CA, USA). Primers were designed to amplify fragments of approximately 100 bp for all genes of interest, including *rpoD*, which was used as an internal control for normalization. The *rpoD* gene has been described as an invariant housekeeping gene for *P*. *syringae* [43]. RT-qPCR amplifications were carried out on a ABI PRISM 7300 RT PCR System using a SYBR Green PCR Master Mix (Applied Biosystems, Madrid, Spain). Thermal cycling conditions were as follows: one cycle at 95°C for 10 min; 50 cycles at 95°C for 15 s and 65°C for 1 min; and a final cycle at 95°C for 15 s, 60°C for 1 min, 95°C for 15 s and 60°C for 15 s. The relative gene expression ratio was calculated using the comparative critical threshold (ΔΔC_t_) method [44, 45].

### Swarming motility assays

*PsPto* strains were grown at 28°C for 24 h on KB agar under dark conditions. Cells were resuspended in KB medium to an OD_600_ of 1. Five microliters of the bacterial suspension were spotted onto soft KB agar (KB with 0.5% wt/vol BD Bacto dehydrated agar [ThermoFisher Scientific, Waltham, MA, USA]). Plates were incubated for 16 h at 28°C and 80% relative humidity under dark conditions. Four replicate experiments were performed. Swarm colonies were photographed, the surface area of each colony was quantified using the area selection tool of Adobe Photoshop CS5 v. 12.0 software (Adobe Systems, San Jose, CA, USA) with readings in pixels, and treatment comparisons were made using Fisher’s LSD test (*p-*value < 0.05).

### Tomato infection assays

For the infection assays, 3-week-old tomato plants (*Solanum lycopersicum* cv. Moneymaker) were incubated in a growth chamber at 25°C with a 12-h light (100 μE m^-2^ s^-1^) / 12-h dark cycle and a relative humidity of 60%. Wild-type and mutant bacterial cells were harvested from fresh KB plates and resuspended in 10 mM MgCl_2_ to reach an cell density of 1 x 10^6^ CFU/ml. Silwet L-77 (Lehle Seeds) was added to a final concentration of 0.02% (vol/vol) and plants were inoculated by dipping into the bacterial solution for 30 s. Control plants were dipped in a solution of 10 mM MgCl_2_ with 0.02% (vol/vol) Silwet. Bacterial growth *in planta* was determined 5 days postinoculation (dpi) by collecting samples from infected and control leaves using a cork borer. Leaf disks (0.785 cm^2^; three disks from each independent plant) were homogenized in 10 mM MgCl_2_, and serial dilutions were plated onto KB plates supplemented with the appropriate antibiotics.

### Statistical analysis

All statistical analyses were performed using the software package SPSS Statistics v. 20.0 (SPSS, Chicago, IL, USA).

## Results

### Bioinformatic analysis of *PsPto* RND efflux pumps

To identify putative *PsPto* MDR transporters from the RND family that could play a role in the plant infection process, we first performed a search for candidate RND genes in the genome of *PsPto*. Using the information available in the Pfam database (release 32.0) [36] about the conserved domains that define this protein family (Pfam family PF00873) different HMM profiles were built and used to search against the *PsPto* genome database, as detailed in Materials and Methods. The resulting list of candidate genes is shown in Table 2. Nine putative RND-type efflux systems were found. Three of them, *mexB*, *saxG* and *saxF* (probable orthologs of the Mex systems of *P*. *aeruginosa*) had been previously characterized in *PsPto* [24, 25, 27]. Since the major efflux pumps of *PsPto* were already investigated, we decided to focus our attention on RND transporters evolutionarily distant from MexB. PSPTO_5191 and PSPTO_0820 (38.8% identical to each other) were the RND proteins that showed the lowest sequence identity to MexB, 21.7% and 20.2%, respectively (over their entire length). PSPTO_5191 (named TpsC) has already been studied by Carvajal et al. [46] who suggested it could be involved in the efflux of flavonoid phloretin and *PsPto* virulence in tomato. Sequence comparisons of PSPTO_0820 revealed that orthologs of this protein were well conserved among *Pseudomonas* species that interact with plants, particularly within plant pathogenic pseudomonads (S2 Table). PSPTO_0820 also showed high similarity to RND transporters of the AcrB/AcrD/AcrF family from other bacteria such as *Azotobacter chroococcum* (90% identity) and *Paraburkholderia tuberum* (87% identity), species which can be found in rhizosphere soils or nitrogen-fixing nodules on legume roots, respectively. We then decided to investigate whether PSPTO_0820 had a role in *PsPto*-plant interaction.

**Table 2:** Inventory of predicted MDR transporters from the RND (Pfam PF00873) superfamily encoded in *PsPto* genome.

NCBI ID	NCBI gene description	Notes
RND-type:		
PSPTO_0375	Cation efflux family protein	
* PSPTO_0820	AcrB/AcrD/AcrF family protein	
PSPTO_1308	AcrB/AcrD/AcrF family protein	
PSPTO_2592	Aliphatic isothiocyanate resistance protein SaxG; AcrB/AcrD/AcrF family	Putative ortholog of *P*. *aeruginosa* PAO1 MexD
PSPTO_2755	AcrB/AcrD/AcrF family protein	
PSPTO_3100	Aliphatic isothiocyanate resistance protein SaxF; AcrB/AcrD/AcrF family	Putative ortholog of *P*. *aeruginosa* PAO1 MexF
PSPTO_3302	AcrB/AcrD/AcrF family protein	
PSPTO_4304	Isothiocyanate resistance protein SaxB	Putative ortholog of *P*. *aeruginosa* PAO1 MexB
PSPTO_5191	TpsC transporter	

(*) Gene analyzed in this work.

**Fig 1:**
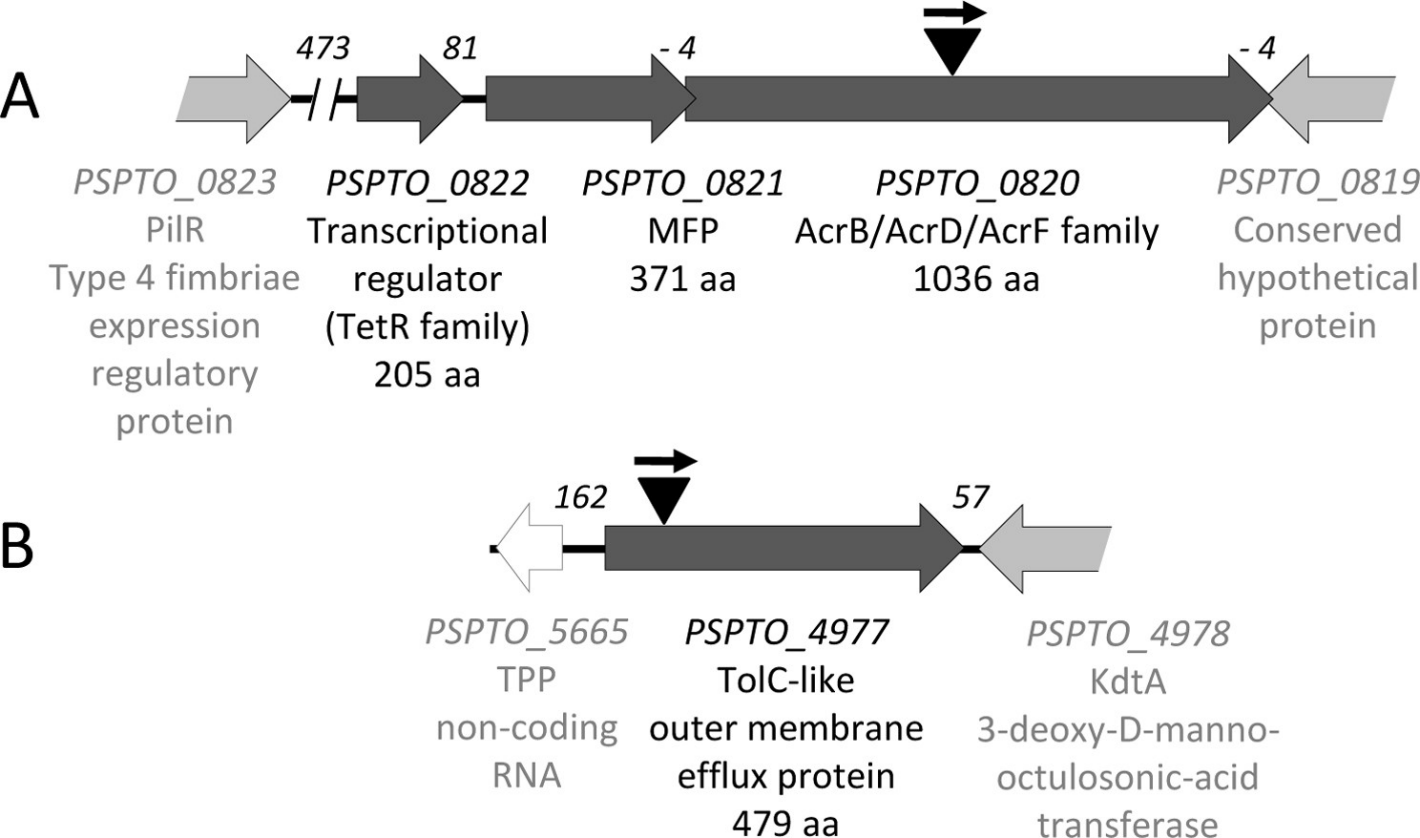
Schematic map of *PsPto* DNA regions altered in the mutants analyzed in this work. A, genomic context of PSPTO_0820 gene. B, genomic context of PSPTO_4977 gene. Arrows indicate the different open reading frames (ORFs) and their transcriptional direction. Closed dark grey arrows indicate candidate genes putatively related with MDR functions; the remaining arrows indicate adjacent ORFs (those showed in light grey are only partially drawn). The white arrow upstream of *PSPTO_4977* gene is annotated as a putative small non-coding RNA gene (*PSPTO_5665*). Below each ORF are indicated the GenBank identifiers, the putative protein products, and their predicted size (in amino acids), as well as the MDR protein family to which they belong. Black triangles indicate the relative positions of the mini-Tn*5gusA*Gm insertions (the small arrow represents the orientation of the promoterless *gusA* gene). Numbers in italic type above the map, in the intergenic regions, indicate the distance (in base pairs) between adjacent genes. Negative numbers denote that the genes overlap by the indicated number of base pairs. B, gene interrupted in PS4977 mutant. Data were obtained from http://www.pseudomonas-syringae.org/pst_home.html.

As mentioned above, the gene *PSPTO_0820* is predicted to encode the inner membrane protein component of a RND family transporter. The gene context of *PSPTO_0820* (Fig 1A) suggests that this gene is part of an operon, since it overlaps 4 bp with the upstream *PSPTO_0821* gene. This gene would encode the MFP component of the system. However, no gene coding for an outer membrane efflux protein was present in the vicinity of these genes. This resembles the situation observed in the *E*. *coli* AcrAB-TolC tripartite efflux pump, where the *acrAB* genes are cotranscribed but the gene encoding the outer membrane component of the pump, *tolC*, is located elsewhere on the chromosome [12]. A search for proteins belonging to the outer membrane efflux protein family (Pfam family PF02321) was carried out in *PsPto* genome. The result of the search is depicted in Table 3. Nine putative candidates were found, of which seven were adjacent to different transporters from the RND or ATP-binding cassette (ABC) families. From the two remaining candidates, PSPTO_4977 was the most similar protein to *E*. *coli* TolC and it was well-conserved within the genus (S3 Table). PSPTO_4977 showed 32% identity to TolC from *E*. *coli* DH1 and 33% to TolC (Dda3937_00726) from *D*. *dadantii* 3937 [47]. Analysis of the deduced amino acid sequence of *PSPTO_4977* gene with the SignalP 4.1 Server (CBS, Technical University of Denmark) predicts (with a discrimination score of 0.884, far over the threshold score of 0.57) the presence of a signal peptide at its amino terminus, as expected for this class of proteins (with a potential peptide cleavage site between amino acids 21 and 22) (S2 Fig). *PSPTO_4977* is a monocistronic gene (Fig 1B) and, apart from a MDR gene of the SMR family (*PSPTO_4979*), no efflux pump coding genes from the RND, major facilitator superfamily (MFS) or ABC family were found in close proximity.

**Table 3:** Prediction of proteins belonging to the outer membrane efflux family (Pfam PF02321) in *PsPto* genome.

NCBI ID	NCBI gene description^*a*^
**PSPTO_0377**	metal ion efflux outer membrane protein ^1^
PSPTO_1217	outer membrane efflux protein
**PSPTO_2158**	outer membrane efflux protein ^2^
**PSPTO_2756**	outer membrane efflux protein ^1^
**PSPTO_3101**	outer membrane efflux protein ^1^
**PSPTO_3328**	ABC transporter permease; TliF ^2^
**PSPTO_3621**	outer membrane efflux protein ^2^
**PSPTO_4305**	outer membrane efflux protein ^1^
* PSPTO_4977	outer membrane efflux protein TolC, putative

(*) Gene analyzed in this work

^*a*^ Genes in bold type, outer membrane efflux genes located adjacent to transporters from the (1) RND protein family and (2) ABC protein family.

Using an arrayed transposon mutant library of *PsPto*, we isolated two mutants in *PSPTO_0820* and *PSPTO_4977* genes (named PS0820 and PS4977, respectively). A schematic view of the transposon location in the disrupted genes is shown in Fig 1. We then proceeded to characterize these two strains.

### PS0820 and PS4977 mutants exhibit increased sensitivity to plant antimicrobials

To test a possible influence of the mutations on the overall fitness of *PsPto*, the growth rates of the different mutant strains in liquid KB medium were compared to that of the wild-type strain. Doubling times of PS0820 and PS4977 mutant strains during the exponential growth phase were similar (in the range of 90 to 95 min) to that of the wild-type strain (90 min), indicating no major defects caused by the mutations, at least under our experimental conditions. Then, phenotype of tolerance/sensitivity of the different *PsPto* mutants to a number of antimicrobial compounds, which affected the growth of the wild-type strain, was examined.

Determination of MICs of antibiotics was performed using E-test strips (see Materials and Methods). Antibiotics analyzed were ampicillin, chloramphenicol, colistin, erythromycin, sulfamethoxazole and tetracycline. Among the mutants, only PS4977 mutant was slightly more susceptible (≤ 2-fold difference in MIC) to the antibiotics erythromycin and tetracycline (S4 Table). Susceptibility to other antimicrobial compounds, such as detergents and dyes (acriflavin, crystal violet, deoxycholate, and rhodamine) was further tested by a microtiter broth dilution method. No significant differences were found when compared to those of the wild-type strain (S3 Fig).

**Fig 2:**
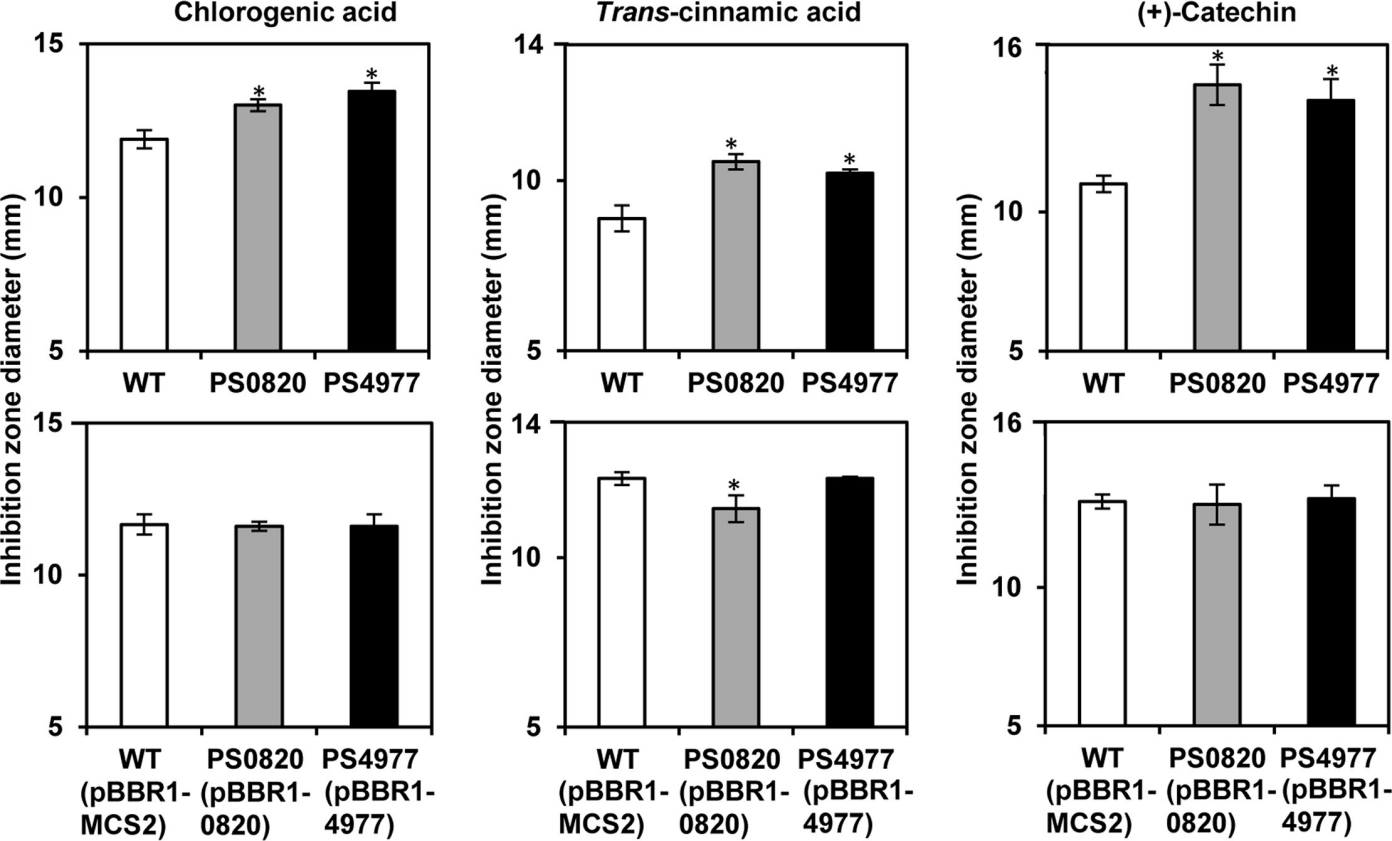
Antimicrobial susceptibility assays. Bacterial cells (*PsPto*, the MDR mutants or the mutant complemented strains) were inoculated onto LB plates in the presence of different plant antimicrobial solutions (0.5 M) deposited into a well at the center of the plate. The diameter of the growth inhibition halo was measured after 24 h incubation at 28°C. Data represent the means and standard errors of at least nine independent replicates. Asterisks indicate significant differences (p<0.05) between the mean values of the wild-type strain in comparison with those of the MDR mutants, or between the wild-type strain (harbouring the pBBR1MCS-2 empty vector) and the corresponding complemented mutants.

With the aim of elucidating the putative physiological role(s) of different efflux pumps in the context of plant-bacteria interaction, we tested for possible differences in sensitivity between the wild-type and each of the mutants to a number of plant antimicrobial compounds from the alkaloid and phenylpropanoid classes. Due to the low solubility of these compounds in water, we carried out growth inhibition plate assays on KB medium using DMSO as a solvent. DMSO by itself had no detectable effects on the growth of the bacteria in these conditions. The different mutants exhibited a phenotype similar to that of the wild-type strain to the alkaloids berberine, plumbagin, and rhein, to the flavonoids genistein, morin, naringenin, phloretin, phloridzin, and quercetin, and to other phenylpropanoids, such as the coumarin esculetin, and the hydroxycinnamic acid derivatives caffeic acid and *trans*-ferulic acid (S4 Fig). However, both PS0820 and PS4977 mutants were significantly more susceptible than the parental strain to *trans*-cinnamic acid, the hydroxycinnamic acid derivative CGA, and to the flavonoid (+)-catechin (Fig 2).

We also tested whether the complementation of the mutations by the corresponding wild-type copies of the genes could restore the resistant phenotype of these strains. To perform the complementation analysis, the MDR genes were cloned into the medium-copy-number vector pBBR1MCS-2 (Table 1) and transferred to the mutant strains. The wild-type genes were able to complement the susceptibility phenotype to plant antimicrobials exhibited by the corresponding mutants (Fig 2). In the case of *trans*-cinnamic acid, the complemented PS0820 mutant was more resistant to this compound than the wild-type strain harbouring the pBBRMCS-2 empty vector (Fig 2).

### Influence of plant antimicrobials on the MDR gene expression

**Fig 3:**
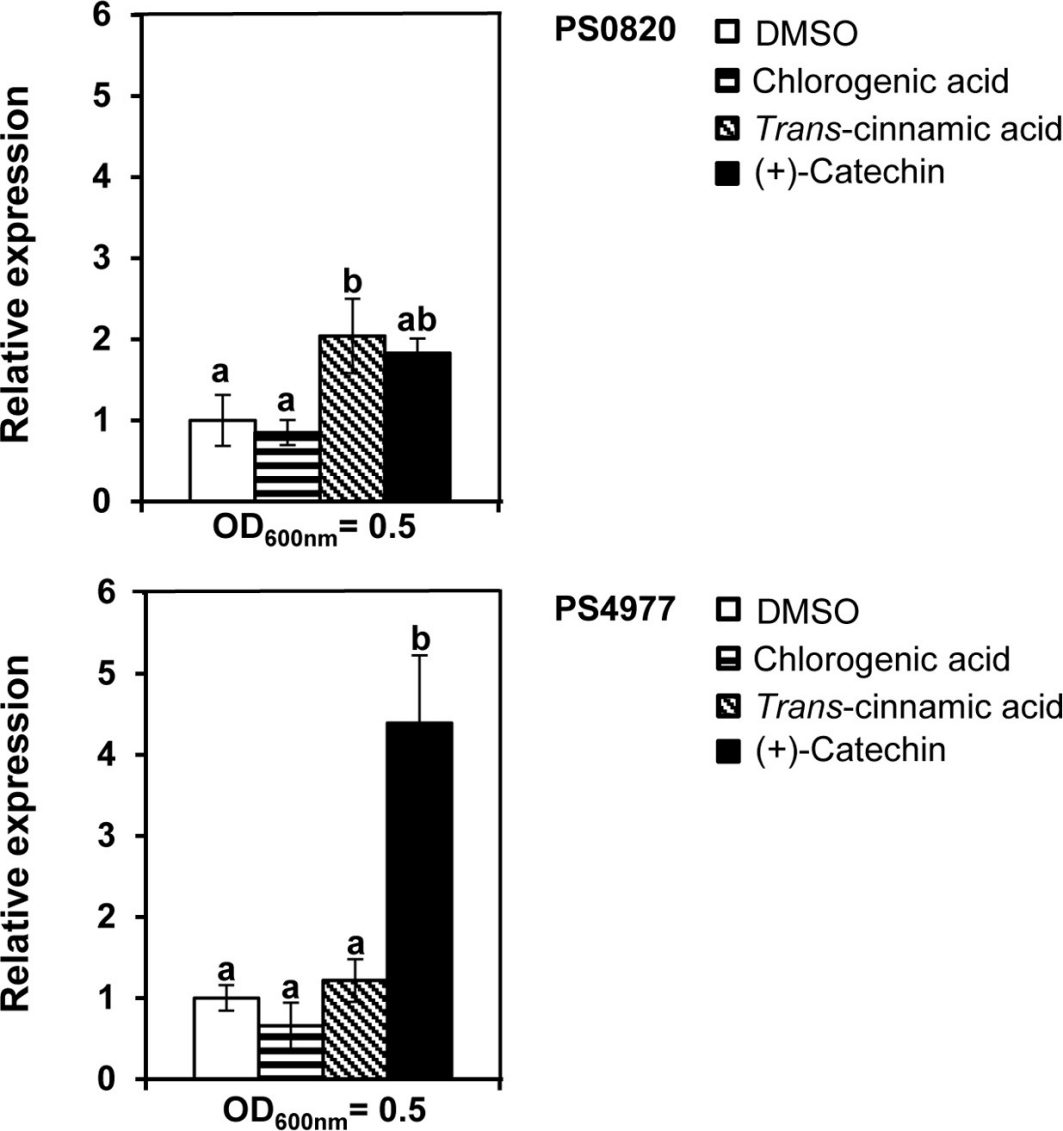
Effect of plant antimicrobial compounds on MDR gene expression. Differential gene expression was evaluated by RT-qPCR after *PsPto* cells were grown in the presence of 0.5 mM of the indicated compounds (solved in DMSO) or DMSO. Cell samples were harvested at the mid exponential growth phase (OD_600_ = 0.5). Bars represent relative expression changes of target genes in cells grown in the presence of antimicrobial compounds with respect to DMSO-grown cells. Data represent means and standard errors of three replicates, with different letters indicating significant differences among treatments (*p* < 0.05, Fisher’s LSD test).

To analyze whether the putative substrates of these efflux pumps affected the *in vivo* gene expression of these genes, total RNA was isolated from cells grown in fructose M9 minimal medium in the absence or the presence of 0.5 mM of the following compounds: CGA, *trans*-cinnamic acid and (+)-catechin. These antimicrobial concentrations did not affect bacterial growth. Addition of CGA to the cell cultures did not induce, in any case, MDR gene expression above the levels reached by the control cultures with DMSO (Fig 3). Presence of *trans*-cinnamic acid resulted in a 2-fold increase in the expression of *PSPTO_0820* gene (Fig 3). (+)-Catechin increased expression of both genes (about 2-fold for *PSPTO_0820*, and more than 4-fold for *PSPTO_4977*) (Fig 3).

### PS0820 and PS4977 mutants are affected in plant colonization

**Fig 4:**
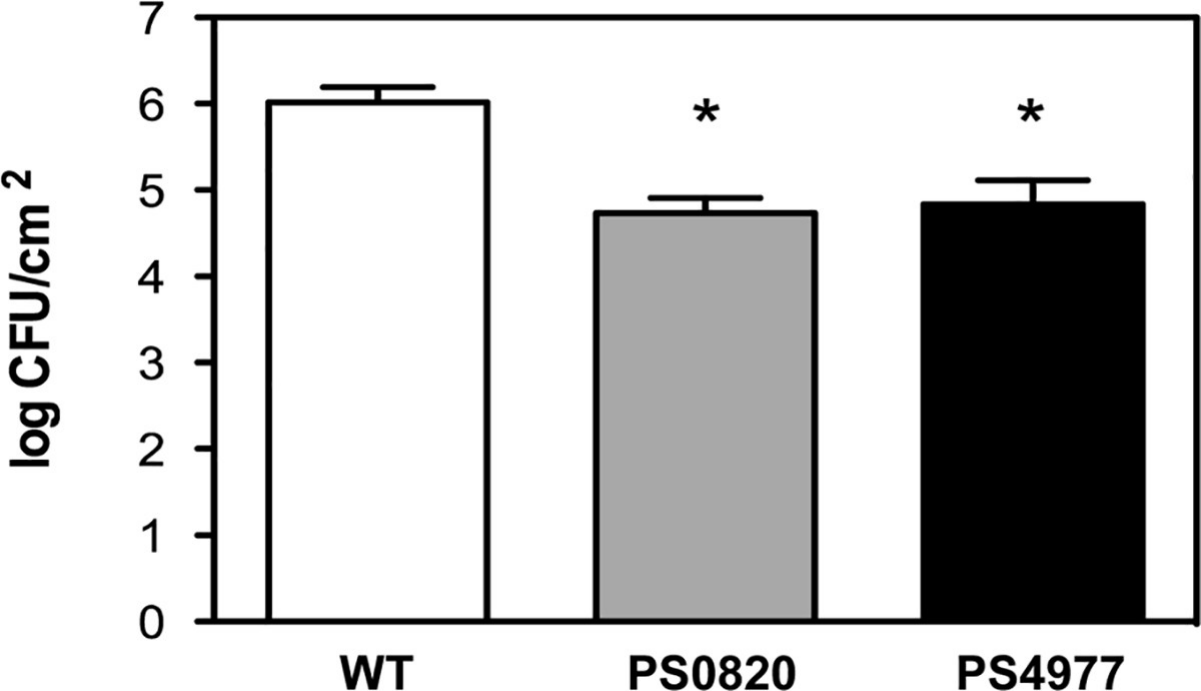
Bacterial growth of *PsPto* and the mutant strains inoculated in tomato plants. Bacterial population was determined at 5 dpi. Data represent the average of 5 independent experiments with the standard error. Asterisks indicate significant differences between means (p<0.05) in comparison with the values of the wild-type strain.

The ability of the wild-type and the mutant strains to colonize and survive in tomato plants was compared in infection assays by counting bacterial populations 5 days after dip-inoculation. Wild-type strain reached population densities averaging about 1 x 10^6^ CFU/cm^2^ of leaf tissue. PS0820 and PS4977 mutants showed more than a 10-fold decrease in their population sizes, with averages of 5.4 x 10^4^ CFU/cm^2^ and 6.9 x 10^4^ CFU/cm^2^ of leaf, respectively (Fig 4). These results suggest impaired plant colonization abilities for both *PsPto* mutants compared to the wild-type.

### *PsPto* MDR mutants display normal swarming motility

**Fig 5:**
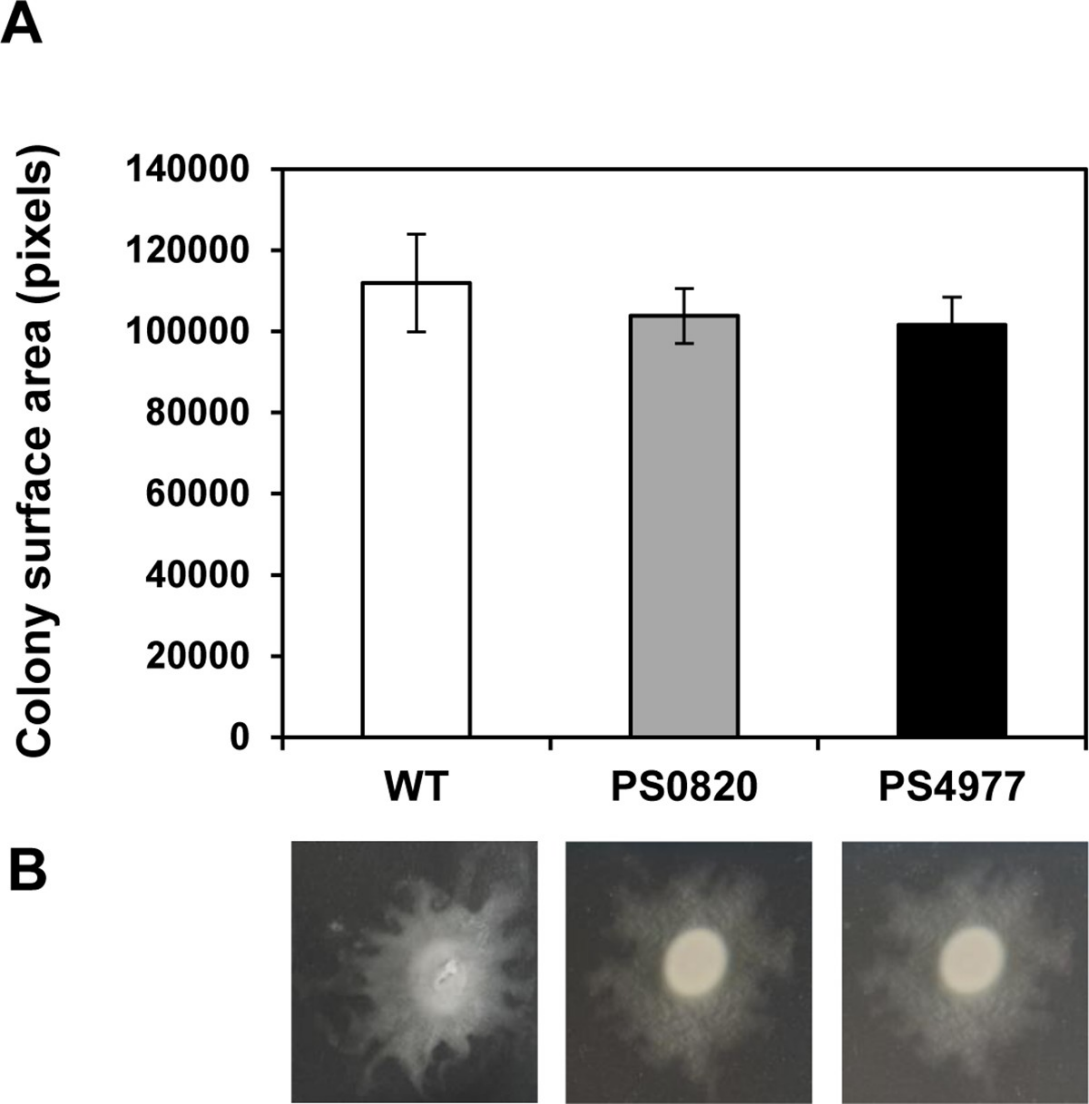
Swarming motility assays. The ability of wild-type and *PsPto* MDR mutant strains to swarm on soft agar was assessed. Five microliters of a bacterial suspension in KB medium (at 1 OD_600_) were spotted onto soft KB agar (0.5% [wt/vol]) plates and incubated for 16 h at 28°C and 80% relative humidity under dark conditions. A, The surface area of each swarm colony was quantified based on pixel counts in a digitized image. Data represent the means and standard errors of four replicates (*p* < 0.05, Fisher’s LSD test). B, Photographs of representative swarm colonies.

Swarming motility contributes to *P*. *syringae* ability to invade leaves and cause disease [48]. A recent report shows that *acrD* inactivation in *Salmonella enterica* causes a significant reduction on swarming motility, suggesting the potential implication of efflux pumps in bacterial motility [49]. Considering the importance of motility in the *PsPto* colonization process, we decided to investigate the swarming ability of *PsPto* MDR mutants. When swarming motility of the mutant strains was tested on KB plates with 0.5% agar, it was similar in all cases to that of the wild-type strain (Fig 5).

## Discussion

To establish a successful infection, phytobacteria must be able to withstand several plant host defense mechanisms. Among the first barriers that *PsPto* has to overcome when entering the plant is the presence of plant antimicrobial compounds. In this context, the expression of bacterial MDR efflux systems is a key factor for the survival of the pathogen in the apoplastic space. Although there are a few studies on the function of MDR transporters in plant-bacterial interactions, knowledge about *P*. *syringae* MDR sytems is very limited.

In this work, we investigated the phenotypes caused by mutations in two different *PsPto* genes related to multidrug efflux, to elucidate their roles in the plant infection process. *PSPTO_0820* is predicted to code an inner membrane multidrug transporter and *PSPTO_4977* codes for an outer membrane efflux protein with similarity to TolC family proteins. When the resistance/susceptibility phenotypes of the wild-type and mutant strains to various toxic compounds, including plant antimicrobials, were compared, the main differences were found for some compounds of the phenylpropanoid group. Phenylpropanoids are of special interest since it has been reported that in tomato plants, an increased expression of genes from the phenylpropanoid pathway is associated with bacterial infection, leading to the accumulation of some of these compounds in the infected tissues [7–9]. S5 Fig summarizes the major biosynthetic routes to the various classes of phenylpropanoid compounds. Hydroxycinnamic acids and flavonoids are ubiquitous in higher plants, although the specific substitution patterns can vary depending on plant species [4, 50].

Both mutants were more susceptible than the wild-type strain to CGA and (+)-catechin (Fig 2). CGA (5-*O*-caffeoyl-quinic acid) is an intermediate of the lignin biosynthetic pathway [51] and, together with the flavonoid glycoside rutin, it is one of the most abundant phenolics constitutively expressed in tomato foliage [9]. Interestingly, CGA was found to increase more than 2-fold its concentration in tomato leaves 4 hours after *PsPto* infiltration, reaching a 4-fold increase 48 h after the challenge, as demonstrated by López-Gresa and coworkers by using whole tissue extracts [9]. These authors also showed through the exogenous addition of CGA that this compound induced the *in vitro* expression of tomato defense-related genes, suggesting that CGA could play a defensive-role in the tomato-*PsPto* interaction [9]. In addition, CGA overproduction in tomato was reported to improve its resistance to infection by *P*. *syringae* pv. tomato T1 [52]. It is noteworthy that it has been recently shown that also in tobacco plants there is a specific accumulation of chlorogenic acid isomers in the apoplastic compartment in response to *P*. *syringae* pv. syringae or tabaci infections [53]. Apart from its antibiotic properties, it has been evidenced in different studies that CGA is an ubiquitous phenolic acid that can exhibit multiple modes of protection during host defense [54, 55]. Villarino and coworkers [56] reported that CGA in immature peach inhibited infection by *Monilinia laxa* by interfering with fungal melanin biosynthesis, although *M*. *laxa* growth was not affected by this compound. CGA was also recently shown to reduce *Alternaria alternata* colonization of tomato fruit by inhibiting the synthesis of the toxin alternariol [57]. Our results suggest that the MDR systems inactivated in PS0820 and PS4977 mutants could play a direct role in resistance of *PsPto* to CGA, presumably serving as export pumps. This compound would indeed function as an antimicrobial agent in tomato defense response against *PsPto*, without excluding additional roles as a signal molecule or as an inhibitor of virulence factors. Apart from CGA and rutin, different studies using the tomato-*PsPto* pathosystem have also detected the accumulation of a number of hydroxycinnamic acid amides (HCAA), i.e., hydroxycinnamic acids such as *p*-coumaric and ferulic conjugated to alkaloids (tyramine and octopamine) or to dopamine or noradrenaline [7, 8]. We have not specifically used these compounds in our assays, because most of them are not commercially available, but we tested two other hydroxycinnamic acid derivatives, caffeic acid and *trans*-ferulic acid, and their precursor, *trans*-cinnamic acid. *PsPto* mutants did not show more sensitivity against the two former phenolics but were more susceptible than the wild-type to *trans*-cinnamic acid (Fig 2). It is well known that certain modifications of the phenylpropanoid carbon skeleton, such as prenylation, glycosylation, methoxylation, and others, have a significant impact on their antimicrobial activity, sometimes increasing it and at other times decreasing it [58]. This could explain in part the observed differences in sensitivity against these similar compounds. PS0820 and PS4977 mutants also showed increased susceptibility to the flavanol (+)-catechin (Fig 2). This compound and its derivatives can damage bacterial membranes by directly penetrating them and disrupting their barrier function, or by promoting membrane fusion [58]. It is important to highlight that these susceptibility phenotypes were observed despite the fact that MexAB-OprM has a major role in the efflux of (+)-catechin in *PsPto*, since the *mexA* mutant strain shows a much higher growth inhibition halo in the presence of (+)-catechin under equivalent experimental conditions [25]. In fact, it is very probable that the real impact of *PSPTO_0820* and *PSPTO_4977* mutations is masked by overlapping substrate profiles of MexAB and other important *PsPto* MDR transporters, such as SaxF or SaxG. The amount of plant antimicrobials required for efficient bacterial growth inhibition in *in vitro* assays is usually very high. There is no available data regarding the concentration of these compounds that bacteria find in plant tissues during the interaction. However, it is important to denote that plants defend their vascular systems with phenolic-storing cells stationed along the xylem [59]. When these sentinel cells release phenolics in response to pathogen infection, antimicrobial concentrations may be locally high [60]. In addition, it is known that bacterial susceptibility to plant antimicrobials is usually higher in the presence of compound mixtures (the plant environment) than when a single chemical is present [61, 62].

*PSPTO_0820* seems to be part of a bicistronic operon, which lacks the gene encoding the outer membrane component. It could be tempting to speculate that PSPTO_4977 could act as an outer membrane channel for this MDR system. However, our current results do not allow us to undoubtedly reach that conclusion. On the other hand, PSPTO_4977 could have a role similar to the one played by TolC in *E*. *coli*, where this protein functions in conjunction with multi-component transporters belonging to different MDR families [12]. However, if this would be the case, one would expect a higher susceptibility phenotype after its inactivation. Further studies would be needed to elucidate these questions.

Most RND efflux pumps are subjected to tight regulatory controls to maintain low levels of expression, since overexpression of integral membrane proteins that utilize proton-motive force is deleterious for the cell [63]. In this sense, it is very common that RND efflux pump genes have a local regulator gene in its vicinity. This would be in accordance with the presence, 81 bp upstream of *PSPTO_0821*, of a putative transcriptional regulator encoded by *PSPTO_0822* gene (Fig 1A). This protein belongs to the TetR family of transcriptional regulators, which usually act as repressors [64].

We also analyzed by RT-qPCR assays whether the expression of the MDR genes increased in the presence of any of the phenylpropanoids CGA, *trans*-cinnamic acid and (+)-catechin (Fig 3). A rise in expression was observed for both genes in the case of (+)-catechin. With the current data we cannot determine if the increase in expression is directly or indirectly mediated by this phenylpropanoid, but the results support the idea that (+)-catechin is indeed a substrate for these two systems. With the remaining compounds, a change in expression was only observed for *PSPTO_0820* in the presence of *trans*-cinnamic acid. However, the fact that the putative substrates of these MDR pumps do not induce the efflux pump expression is not an uncommon phenomenon. Even in cases where there is a local regulator that responds to a wide spectrum of structurally dissimilar compounds, the effector profile of the regulator is usually narrower than the substrate range of the MDR that controls [18, 25]. For example, in *PsPto*, MexAB expression is not induced in the presence of (+)-catechin although this compound is a substrate for this efflux pump [25].

The ability of the wild-type and the mutant strains to colonize tomato was also evaluated. PS0820 and PS4977 showed a significant decrease in their colonization efficiency (Fig 4), which is in the same order of magnitude than those observed for other *PsPto* MDR mutants [24, 25]. The results demonstrate that both proteins play important roles in the initial stages of the tomato plant colonization by *PsPto*. Finally, since *PsPto* mutants displayed similar swarming phenotypes, it can be deduced that the differences in plant colonization between the MDR mutants and the wild-type strain are not attributable to an indirect effect caused by swarming motility defects. The decreased colonization ability is probably a consequence of the reduced capacity of these bacteria to survive and multiply within the host apoplast in the presence of certain antimicrobial plant compounds.

In short, the present work evidences the involvement of PSPTO_0820 and PSPTO_4977 proteins in the resistance to certain plant antimicrobials from the phenylpropanoid family. The results suggest that CGA, (+)-catechin, and *trans*-cinnamic acid are expelled by these systems, and both *PsPto* genes are important for the colonization of the tomato plant. The putative overlapping substrate profile with other *PsPto* efflux pumps, as it is the case of MexAB and (+)-catechin, would explain why the mutations in these two genes do not show more pronounced differences in antimicrobial sensitivity compared to the wild-type. It seems clear that in *PsPto* there is not only a single MDR system as the sole factor responsible for the resistance to certain plant antimicrobial compounds. In fact, it has been shown that in bacteria, the overlapping specificities of MDR transporters ensure protection against a wide spectrum of toxic compounds, providing at the same time robustness in the response to environmental stresses and conferring evolvability to the microorganisms [65].

## Supporting information

S1 Table Primers used in this work. (PDF)

S2 Table Conservation of PSPTO_0820 within the *Pseudomonas* genus. (PDF)

S3 Table Conservation of PSPTO_4977 within the *Pseudomonas* genus. (PDF)

S4 Table Minimum inhibitory concentration (MIC) values (in μg/ml) of *PsPto* wild-type and MDR mutant strains to different antibiotic compounds. (PDF)

S1 Fig Southern-blot analysis of MDR mutant strains. (PDF)

S2 Fig Analysis of the deduced amino acid sequence of *PSPTO_4977* gene with SignalP 4.1 Server. (PDF)

S3 Fig Effect of antimicrobial compounds on MDR mutant growth as compared to the *PsPto* wild-type. (PDF)

S4 Fig Susceptibility of *PsPto* wild-type and MDR mutants to other alkaloids and phenylpropanoids. (PDF)

S5 Fig Schematic view of the main biosynthetic pathways leading to phenylpropanoid products in plants. (PDF)
