# Supplementary material for: The Pseudomonas syringae pv. tomato DC3000 PSPTO_0820 multidrug transporter is involved in resistance to plant antimicrobials and bacterial survival during tomato plant infection
Source: PLoS One. 2019 Jun 25;14(6):e0218815. doi: 10.1371/journal.pone.0218815 (PMC6592562; doi:10.1371/journal.pone.0218815)
Supplement: S1 Table — (PDF) [file pone.0218815.s001.pdf]

S1 Table. Primers used in this work

Primer	Sequence 5'→3' [a]	Restriction site [b]
For complementation:		
PS0820-5'EcoSD	TTGAATTCagg**ag**gCGCGCCCATGAGCGAAGG	EcoRI
PS0820-3'Sac	TTGAGCTCATGTCTGCACCCGAGCC	SacI
PS4977-5'Eco-n	TTGAATTCTTCATGAACAGGGAATGCC	EcoRI
PS4977-3'Sac	TTGAGCTCGTTCCTCACTCCAGCG	SacI
For mutant screening:		
PS0820-5'	CACCACCATTACTGTGAAG	
PS0820-3'	ATAGAGGGTATTACTGTGAAG	
PS4977-5'	GGTTGCGGATTTATTGTCGGAG	
PS4977-3	ACCTGTTCAACTTCCTCGAGAT	
GUSR2	TTGGGGTTTCTACAGGACGTAACAT	
For pAGM1 construction:		
Gm5-Sp	ACAGCATGCGACGCACACCGTGGAAC	SphI
Gm3-Bss	TTGGCGCGCGGCGTTGTGACAATTTACC	BssHII
For qRT-PCR:		
rpoDFw	CGGCATCGACATGAATACCG	
rpoDRev	GCGCAGTGCCTTGGCTTC	
0820RTNF	GGTGACGAGGCCATGACGC	
0820RTNRev	GCAGCGATTCGGCGTCAC	
4977RTF	CAGGTCCGCGACCTTGATG	
4977RTRev	GCACCGTCAGTTGGTTCGC	

[a] The improved Shine-Dalgarno sequence in primer PS0820-5'EcoSD is indicated in lowercase (bases showed in boldface are those that have been modified). The predicted start codon for the *PSPTO_0820* gene is underlined.

[b] Recognition sites for the restriction enzymes included in the primers to facilitate cloning are indicated.
