# Supplementary material for: The Pseudomonas syringae pv. tomato DC3000 PSPTO_0820 multidrug transporter is involved in resistance to plant antimicrobials and bacterial survival during tomato plant infection
Source: PLoS One. 2019 Jun 25;14(6):e0218815. doi: 10.1371/journal.pone.0218815 (PMC6592562; doi:10.1371/journal.pone.0218815)
Supplement: S2 Table — (PDF) [file pone.0218815.s002.pdf]

S2 Table. Conservation of PSPTO_0820 within the *Pseudomonas* genus

Strain name	Strain accesion	Gene	PercIdent	E-value
Pseudomonas syringae pv. tomato str. DC3000	SAMN02604017	PSPTO_0820	100.00%	0
Pseudomonas syringae pv. tomato NCPPB 1108	SAMN02472091	PTO1108_RS11830	99.71%	0
Pseudomonas syringae pv. lachrymans M302278PT	SAMN02471324	PLA106_RS07355	99.61%	0
Pseudomonas syringae pv. tomato K40	SAMN02472092	PTOK40_RS15810	99.61%	0
Pseudomonas syringae pv. tomato Max13	SAMN02472093	PTOMAX13_RS11920	99.61%	0
Pseudomonas syringae pv. tomato T1	SAMN02472090	PSPTOT1_RS05375	99.61%	0
Pseudomonas syringae pv. actinidiae ICMP 9853	SAMN02471921	JN853_RS03690	99.02%	0
Pseudomonas syringae pv. actinidiae ICMP 18884	SAMN02727983	IYO_RS24940	98.92%	0
Pseudomonas syringae pv. morsprunorum M302280PT	SAMN02471318	PSYMP_RS05200	98.83%	0
Pseudomonas syringae pv. tabaci ATCC 11528 [TSL]	SAMN00002878	C1E_RS28275	96.48%	0
Pseudomonas amygdali pv. lachrymans	SAMN06606132	B5U27_RS03860	96.38%	0
Pseudomonas syringae pv. aesculi 2250	SAMN02471154	IC51_RS0126395	96.38%	0
Pseudomonas syringae pv. aesculi NCPPB3681	SAMN00002867	PSAESCULI_RS18490	96.38%	0
Pseudomonas syringae pv. lachrymans M301315	SAMN02471323	PLA107_RS28500	96.38%	0
Pseudomonas savastanoi NCPPB 3335	SAMN02471367	PSA3335_RS23890	96.09%	0
Pseudomonas syringae pv. glycinea B076	SAMN02471826	PSGB076_RS24395	96.09%	0
Pseudomonas syringae pv. phaseolicola 1448A	SAMN02603162	PSPPH_RS03740	95.89%	0
Pseudomonas syringae CC1557	SAMN02471566	N018_RS12640	95.11%	0
Pseudomonas salegens	SAMN05216210	BLU07_RS14595	87.08%	0
Pseudomonas cichorii JBC1	SAMN02641561	BH81_RS10460	82.51%	0
Pseudomonas cedrina	SAMN04490182	BLU91_RS09230	82.22%	0
Pseudomonas mediterranea	SAMN05216476	BLU23_RS19110	81.95%	0
Pseudomonas corrugata	SAMN04490183	BLU14_RS11340	81.50%	0
Pseudomonas sp. GR 6-02	SAMN03701341	PGR6_RS11495	81.24%	0
Pseudomonas fuscovaginae	SAMN05216581	BLW67_RS19095	81.04%	0
Pseudomonas asplenii	SAMN05216598	BLU37_RS22920	80.94%	0
Pseudomonas frederiksbergensis	SAMN06102480	PFAS1_RS22245	80.78%	0
Pseudomonas moraviensis	SAMN04490196	BLU71_RS10145	80.72%	0
Pseudomonas rhodesiae	SAMN04490209	BLU13_RS13570	80.57%	0
Pseudomonas granadensis	SAMN05216579	BLU52_RS10175	80.53%	0
Pseudomonas thivervalensis	SAMN04490204	BLS29_RS04260	80.36%	0
Pseudomonas mucidolens	SAMN05216202	BLU75_RS10865	80.18%	0
Pseudomonas mandelii JR-1	SAMN02469697	OU5_RS27980	79.86%	0
Pseudomonas syringae pv. syringae HS191	SAMN03267749	PSYRH_RS14880	79.67%	0
Pseudomonas tolaasii	SAMN06579284	B5P22_RS09005	79.55%	0
Pseudomonas lini	SAMN04490191	BLU65_RS18580	79.53%	0
Pseudomonas syringae pv. lapsa	SAMN03774723	ACA40_RS10780	79.38%	0
Pseudomonas veronii	SAMN06076152	PverR02_RS22025	79.37%	0
Pseudomonas syringae 31R1	SAMN05421724	BLV36_RS05825	79.28%	0
Pseudomonas syringae pv. aceris M302273PT	SAMN02471316	PSYAR_RS03535	79.28%	0
Pseudomonas syringae UMAF0158	SAMN04053740	PSYRMG_RS01410	79.28%	0
Pseudomonas syringae pv. syringae B301D	SAMN03267739	PSYRB_RS10735	79.09%	0
Pseudomonas syringae pv. syringae B728a	SAMN02604347	Psyr_2194	79.09%	0
Pseudomonas cerasi	SAMEA3894894	PCPL58_RS17580	78.92%	0
Pseudomonas syringae pv. syringae 642	SAMN02472094	COO_RS0109580	78.80%	0
Pseudomonas rhizosphaerae	SAMN03077633	LT40_RS08250	76.53%	0
Pseudomonas yamanorum	SAMN05216237	BLU46_RS28225	58.01%	0
Pseudomonas orientalis	SAMN04490197	BLU00_RS06070	57.52%	0
Pseudomonas brenneri	SAMN04490181	BLU43_RS04675	57.51%	0
Pseudomonas synxantha	SAMN05216475	BLU48_RS08110	57.38%	0
Pseudomonas trivialis	SAMN03699826	AA957_RS01255	57.31%	0
Pseudomonas libanensis	SAMN04490190	BLQ22_RS02765	57.28%	0
Pseudomonas fluorescens PICF7	SAMN03446264	PFLUOLIPICF7_RS20425	57.21%	0
Pseudomonas simiae	SAMN02739889	PS417_RS12220	57.21%	0
Pseudomonas antarctica	SAMN04490179	BLQ27_RS21275	56.82%	0
Pseudomonas resinovorans NBRC 106553	SAMD00061053	PCA10_RS07435	56.23%	0
Pseudomonas sabulinigri	SAMN05216271	BLU26_RS11300	56.14%	0
Pseudomonas koreensis	SAMN05017671	A8L59_RS05415	43.57%	0
Pseudomonas cremoricolorata	SAMN03068908	LK03_RS10730	43.56%	0
Pseudomonas knackmussii B13	SAMEA3139009	PKB_RS05835	43.37%	0
Pseudomonas entomophila L48	SAMEA3138225	PSEEN_RS19605	43.12%	0
Pseudomonas monteilii SB3078	SAMN02641476	X969_RS03950	43.03%	0
Pseudomonas parafulva	SAMN03107785	NJ69_RS02025	42.72%	0
Pseudomonas chlororaphis subsp. aurantiaca	SAMN02953966	JM49_RS24000	42.63%	0
Pseudomonas umsongensis	SAMN04490206	BLU31_RS01845	42.63%	0
Pseudomonas protegens Cab57	SAMD00061024	PPC_RS05980	42.56%	0
Pseudomonas mosselii SJ10	SAMN02470220	O165_RS17060	42.55%	0
Pseudomonas stutzeri	SAMN02692943	UIB01_RS07235	42.49%	0
Pseudomonas aeruginosa	SAMN04455155	PA7790_RS06705	42.47%	0
Pseudomonas alkylphenolica	SAMN02929205	PSAKL28_RS20730	42.45%	0
Pseudomonas citronellolis	SAMN05178539	A9C11_RS24185	42.37%	0
Pseudomonas xanthomarina	SAMN05216535	BLW61_RS08150	42.37%	0
Pseudomonas vancouverensis	SAMN05216558	BLV08_RS01600	42.35%	0
Pseudomonas fragi	SAMN05216594	BLU25_RS09365	42.34%	0

- Plant-pathogen
- Plant-associated
- Animal and Plant-pathogen
- Animal-pathogen

Pseudomonas versuta	SAMN04076495	AOC04_RS15250	42.34%	0
Pseudomonas fulva 12-X	SAMN00713619	PSEFU_RS17605	42.26%	0
Pseudomonas prosekii	SAMN05216222	BLU01_RS07145	42.24%	0
Pseudomonas alcaliphila JAB1	SAMN03784977	UYA_RS17840	42.10%	0
Pseudomonas sihuiensis	SAMN05216363	BLT86_RS12325	42.10%	0
Pseudomonas oryzihabitans	SAMN04158501	APT59_RS21055	42.07%	0
Pseudomonas pohangensis	SAMN05216296	BLT89_RS12520	42.03%	0
Pseudomonas psychrotolerans	SAMN05860868	BJP27_RS15425	42.01%	0
Pseudomonas mendocina S5.2	SAMN02728191	DW68_RS06490	42.00%	0
Pseudomonas poae	SAMN04490208	BLT61_RS09735	41.94%	0
Pseudomonas psychrophila	SAMN04490201	BLU59_RS21735	41.94%	0
Pseudomonas pseudoalcaligenes CECT 5344	SAMEA4532340	BN5_RS05425	41.92%	0
Pseudomonas alcaligenes	SAMN04539664	A0T30_RS07180	41.06%	0
Pseudomonas agarici	SAMN04420121	AWM79_RS15510	41.00%	0
Pseudomonas litoralis	SAMN05216198	BLU11_RS13455	40.96%	0
Pseudomonas oryzae	SAMN05216221	BLT78_RS07280	30.46%	6.00E-24
Pseudomonas guangdongensis	SAMN05216580	BLU22_RS12865	28.68%	6.00E-28
