# Supplementary material for: The Pseudomonas syringae pv. tomato DC3000 PSPTO_0820 multidrug transporter is involved in resistance to plant antimicrobials and bacterial survival during tomato plant infection
Source: PLoS One. 2019 Jun 25;14(6):e0218815. doi: 10.1371/journal.pone.0218815 (PMC6592562; doi:10.1371/journal.pone.0218815)
Supplement: S3 Table — (PDF) [file pone.0218815.s003.pdf]

S3 Table. Conservation of PSPTO_4977 within the *Pseudomonas* genus

strain_name	strain_accesion	gene	percIdent	E-value
Pseudomonas syringae pv. tomato str. DC3000	SAMN02604017	PSPTO_4977	100.00%	0
Pseudomonas syringae pv. lachrymans M302278PT	SAMN02471324	PLA106_RS24305	100.00%	0
Pseudomonas syringae pv. tomato K40	SAMN02472092	PTOK40_RS08850	100.00%	0
Pseudomonas syringae pv. tomato Max13	SAMN02472093	ᐳTOMAX13_RS1104(	100.00%	0
Pseudomonas syringae pv. tomato NCPPB 1108	SAMN02472091	PTO1108_RS14670	100.00%	0
Pseudomonas syringae pv. tomato T1	SAMN02472090	PSPTOT1_RS19955	100.00%	0
Pseudomonas syringae pv. actinidiae ICMP 18884	SAMN02727983	IYO_RS25395	99.79%	0
Pseudomonas syringae pv. actinidiae ICMP 9853	SAMN02471921	JN853_RS27815	99.79%	0
Pseudomonas syringae pv. morsprunorum M302280P	SAMN02471318	PSYMP_RS22355	99.79%	0
Pseudomonas syringae CC1557	SAMN02471566	N018_RS22895	97.08%	0
Pseudomonas syringae 31R1	SAMN05421724	BLV36_RS23690	96.03%	0
Pseudomonas syringae UMAF0158	SAMN04053740	PSYRMG_RS18755	96.03%	0
Pseudomonas amygdali pv. lachrymans	SAMN06606132	B5U27_RS02870	95.82%	0
Pseudomonas syringae pv. glycinea B076	SAMN02471826	PSGB076_RS04950	95.82%	0
Pseudomonas syringae pv. lachrymans M301315	SAMN02471323	PLA107_RS07905	95.82%	0
Pseudomonas syringae pv. phaseolicola 1448A	SAMN02603162	PSPPH_RS02705	95.82%	0
Pseudomonas syringae pv. syringae 642	SAMN02472094	COO_RS0118900	95.82%	0
Pseudomonas cerasi	SAMEA3894894	PCPL58_RS25745	95.62%	0
Pseudomonas savastanoi NCPPB 3335	SAMN02471367	PSA3335_RS25650	95.62%	0
Pseudomonas syringae pv. aesculi 2250	SAMN02471154	IC51_RS0103610	95.62%	0
Pseudomonas syringae pv. aesculi NCPPB3681	SAMN00002867	PSAESCULI_RS1081!	95.62%	0
Pseudomonas syringae pv. lapsa	SAMN03774723	ACA40_RS02780	95.62%	0
Pseudomonas syringae pv. tabaci ATCC 11528 [TSL]	SAMN00002878	C1E_RS04095	95.62%	0
Pseudomonas syringae pv. aceris M302273PT	SAMN02471316	PSYAR_RS15665	95.41%	0
Pseudomonas syringae pv. syringae B728a	SAMN02604347	Psyr_0543	95.41%	0
Pseudomonas syringae pv. syringae HS191	SAMN03267749	PSYRH_RS02695	95.41%	0
Pseudomonas syringae pv. syringae B301D	SAMN03267739	PSYRB_RS03005	95.20%	0
Pseudomonas cichorii JBC1	SAMN02641561	BH81_RS02595	93.11%	0
Pseudomonas chlororaphis subsp. aurantiaca	SAMN02953966	JM49_RS27140	84.97%	0
Pseudomonas protegens Cab57	SAMD00061024	PPC_RS02755	82.81%	0
Pseudomonas asplenii	SAMN05216598	BLU37_RS04755	82.46%	0
Pseudomonas fuscovaginae	SAMN05216581	BLW67_RS28935	82.25%	0
Pseudomonas lini	SAMN04490191	BLU65_RS02450	82.25%	0
Pseudomonas monteilii SB3078	SAMN02641476	X969_RS22975	82.14%	0
Pseudomonas parafulva	SAMN03107785	NJ69_RS21275	82.14%	0
Pseudomonas cremoricolorata	SAMN03068908	LK03_RS03310	82.08%	0
Pseudomonas agarici	SAMN04420121	AWM79_RS19765	81.90%	0
Pseudomonas sp. GR 6-02	SAMN03701341	PGR6_RS02305	81.76%	0
Pseudomonas mosselii SJ10	SAMN02470220	O165_RS19925	81.47%	0
Pseudomonas entomophila L48	SAMEA3138225	PSEEN_RS23015	81.42%	0
Pseudomonas mucidolens	SAMN05216202	BLU75_RS20255	81.22%	0
Pseudomonas mandelii JR-1	SAMN02469697	OU5_RS14910	80.82%	0
Pseudomonas mediterranea	SAMN05216476	BLU23_RS00090	80.79%	0
Pseudomonas corrugata	SAMN04490183	BLU14_RS19785	80.58%	0
Pseudomonas brenneri	SAMN04490181	BLU43_RS25120	80.54%	0
Pseudomonas cedrina	SAMN04490182	BLU91_RS28680	80.38%	0
Pseudomonas prosekii	SAMN05216222	BLU01_RS10055	80.38%	0
Pseudomonas thivervalensis	SAMN04490204	BLS29_RS16890	80.38%	0
Pseudomonas alkylphenolica	SAMN02929205	PSAKL28_RS24165	80.29%	0
Pseudomonas moraviensis	SAMN04490196	BLU71_RS24410	80.29%	0
Pseudomonas versuta	SAMN04076495	AOC04_RS18250	80.29%	0
Pseudomonas granadensis	SAMN05216579	BLU52_RS00910	80.08%	0
Pseudomonas koreensis	SAMN05017671	A8L59_RS02460	80.08%	0
Pseudomonas yamanorum	SAMN05216237	BLU46_RS13650	79.96%	0
Pseudomonas psychrophila	SAMN04490201	BLU59_RS00930	79.87%	0
Pseudomonas synxantha	SAMN05216475	BLU48_RS31090	79.71%	0
Pseudomonas rhodesiae	SAMN04490209	BLU13_RS21805	79.66%	0
Pseudomonas libanensis	SAMN04490190	BLQ22_RS16690	79.50%	0
Pseudomonas veronii	SAMN06076152	PverR02_RS02575	79.50%	0
Pseudomonas frederiksbergensis	SAMN06102480	PFAS1_RS08710	79.32%	0
Pseudomonas orientalis	SAMN04490197	BLU00_RS20025	79.29%	0
Pseudomonas trivialis	SAMN03699826	AA957_RS11610	79.29%	0
Pseudomonas fragi	SAMN05216594	BLU25_RS12535	79.25%	0
Pseudomonas sihuiensis	SAMN05216363	BLT86_RS22455	79.16%	0
Pseudomonas fluorescens PICF7	SAMN03446264	ꟷLUOLIPICF7_RS05ꟷ!	79.08%	0

Plant-pathogen

Plant-associated

Animal and Plant-pathogen

Animal-pathogen

Pseudomonas simiae	SAMN02739889	PS417_RS25400	79.08%	0
Pseudomonas rhizosphaerae	SAMN03077633	LT40_RS14005	79.05%	0
Pseudomonas pseudoalcaligenes CECT 5344	SAMEA4532340	BN5_RS02425	78.81%	0
Pseudomonas tolaasii	SAMN06579284	B5P22_RS28380	78.45%	0
Pseudomonas alcaliphila JAB1	SAMN03784977	UYA_RS02840	78.27%	0
Pseudomonas antarctica	SAMN04490179	BLQ27_RS01025	78.24%	0
Pseudomonas poae	SAMN04490208	BLT61_RS13490	78.24%	0
Pseudomonas fulva 12-X	SAMN00713619	PSEFU_RS20830	78.00%	0
Pseudomonas umsongensis	SAMN04490206	BLU31_RS29430	77.78%	0
Pseudomonas stutzeri	SAMN02692943	UIB01_RS20135	77.53%	0
Pseudomonas resinovorans NBRC 106553	SAMD00061053	PCA10_RS26000	77.43%	0
Pseudomonas mendocina S5.2	SAMN02728191	DW68_RS20280	77.05%	0
Pseudomonas alcaligenes	SAMN04539664	A0T30_RS19280	76.27%	0
Pseudomonas vancouverensis	SAMN05216558	BLV08_RS23040	76.20%	0
Pseudomonas knackmussii B13	SAMEA3139009	PKB_RS26055	75.22%	0
Pseudomonas aeruginosa	SAMN04455155	PA7790_RS29460	74.84%	0
Pseudomonas xanthomarina	SAMN05216535	BLW61_RS19370	74.59%	0
Pseudomonas citronellolis	SAMN05178539	A9C11_RS29110	73.43%	0
Pseudomonas oryzae	SAMN05216221	BLT78_RS07645	67.80%	0
Pseudomonas oryzihabitans	SAMN04158501	APT59_RS17480	63.68%	4,00E-173
Pseudomonas psychrotolerans	SAMN05860868	BJP27_RS09140	63.53%	1,00E-171
Pseudomonas pohangensis	SAMN05216296	BLT89_RS01380	62.86%	0
Pseudomonas guangdongensis	SAMN05216580	BLU22_RS01695	62.36%	0
Pseudomonas salegens	SAMN05216210	BLU07_RS04000	56.79%	8,00E-151
Pseudomonas sabulinigri	SAMN05216271	BLU26_RS07540	56.61%	3,00E-147
Pseudomonas litoralis	SAMN05216198	BLU11_RS12040	56.08%	5,00E-143
