# Supplementary material for: The Pseudomonas syringae pv. tomato DC3000 PSPTO_0820 multidrug transporter is involved in resistance to plant antimicrobials and bacterial survival during tomato plant infection
Source: PLoS One. 2019 Jun 25;14(6):e0218815. doi: 10.1371/journal.pone.0218815 (PMC6592562; doi:10.1371/journal.pone.0218815)
Supplement: S4 Table — (PDF) [file pone.0218815.s004.pdf]

S4 Table. Minimum inhibitory concentration (MIC) values (in µg ml^{-1}) of *PsPto* wild-type and MDR mutant strains to different antibiotic compounds. All assays were carried out in triplicate with identical results.

	DC3000	PS0820	PS4977
Ampicillin	6	6	6
Chloramphenicol	4	4	4
Colistin (polymyxin E)	0.094	0.094	0.094
Erythromycin	4	4	2
Sulfamethoxazole	192	192	192
Tetracycline	0.25	0.25	0.19
