# Supplementary material for: The Pseudomonas syringae pv. tomato DC3000 PSPTO_0820 multidrug transporter is involved in resistance to plant antimicrobials and bacterial survival during tomato plant infection
Source: PLoS One. 2019 Jun 25;14(6):e0218815. doi: 10.1371/journal.pone.0218815 (PMC6592562; doi:10.1371/journal.pone.0218815)
Supplement: S1 Fig — (PDF) [file pone.0218815.s005.pdf]

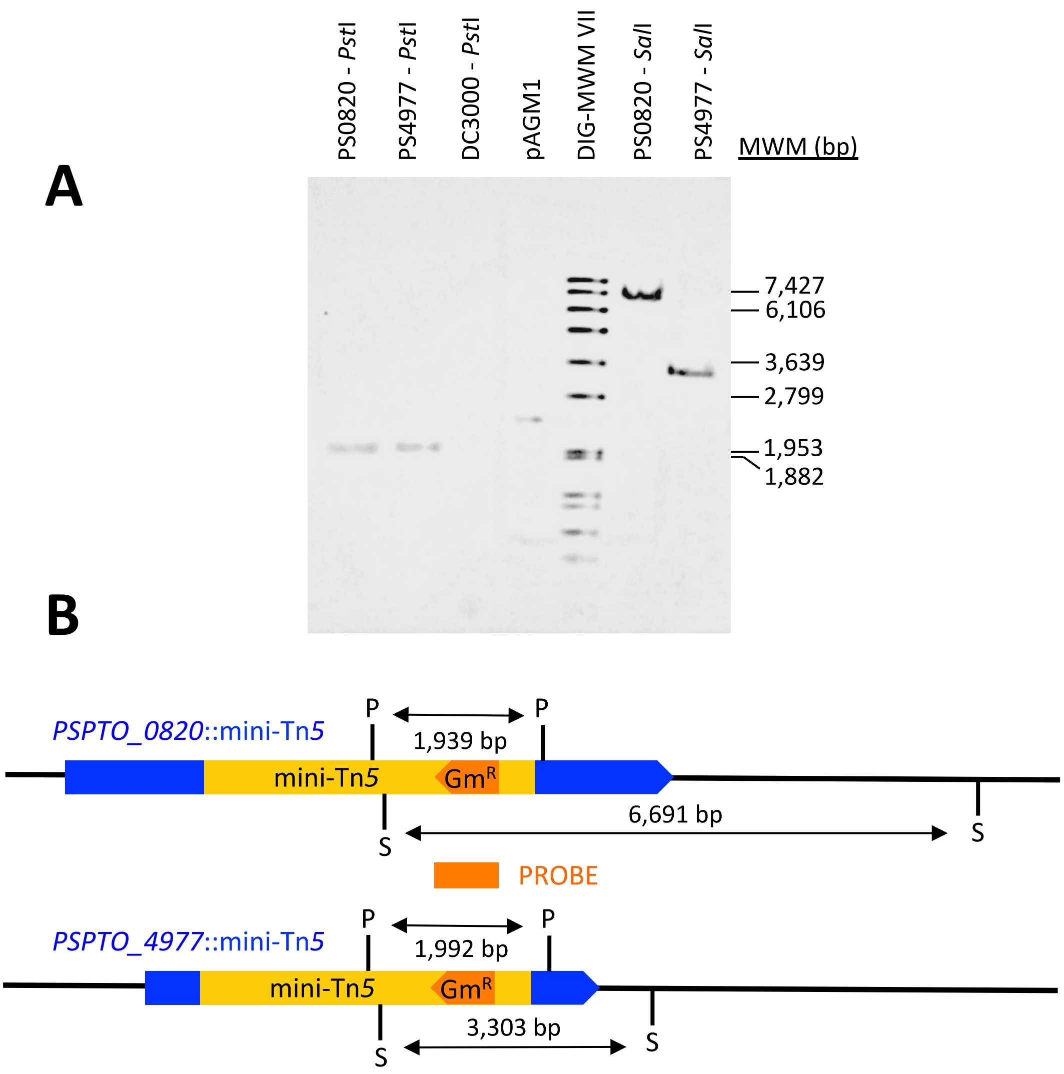

S1 Fig. Southern-blot analysis of MDR mutant strains. Results showed that mutant strains only carry a single insertion of the transposable element in their genomes. A) Genomic mutant DNAs were digested with *Pst*I or *Sal*I. Uncut pAGM1 plasmid (carrying the mini-Tn*5*gusAGm transposon) was included as a positive control and *Pst*I-digested DC3000 genomic DNA was included as a negative control. As the probe, a DNA fragment containing the mini-Tn*5* Gm^R gene was amplified and labeled by PCR with the primers Gm5-Sp and Gm3-Bss (see S1 Table) using digoxigenin-11-dUTP. Hybridized blot was revealed by using an alkaline phosphatase-conjugated anti-digoxigenin antibody and X-phosphate. Bands showing positive hybridization were in agreement with the expected restriction fragments (see panel B). Blue arrows, genes interrupted by the mini-Tn*5* element. P, *Pst*I; S, *Sal*I. Only restriction sites relevant for Southern hybridization are indicated in the maps. The exact positions of the minitransposon insertions were determined by DNA sequencing and are indicated in Table 1.
