# Supplementary material for: The Pseudomonas syringae pv. tomato DC3000 PSPTO_0820 multidrug transporter is involved in resistance to plant antimicrobials and bacterial survival during tomato plant infection
Source: PLoS One. 2019 Jun 25;14(6):e0218815. doi: 10.1371/journal.pone.0218815 (PMC6592562; doi:10.1371/journal.pone.0218815)
Supplement: S2 Fig — (PDF) [file pone.0218815.s006.pdf]

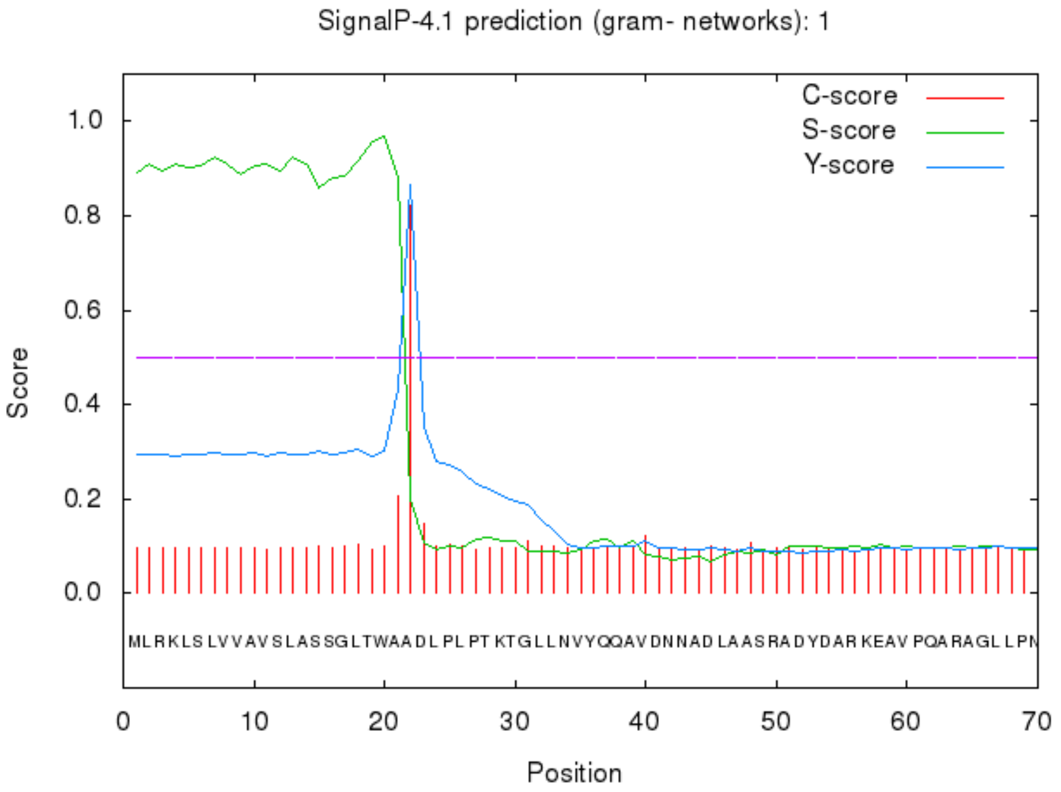

# Measure	Position	Value	Cutoff	signal peptide?
max. C	22	0.823		
max. Y	22	0.864		
max. S	20	0.969		
mean S	1-21	0.906		
D	1-21	0.884	0.570	YES

Name=Sequence SP='YES'
Cleavage site between pos. 21 and 22: TWA-AD
D=0.884 D-cutoff=0.570
Networks=SignalP-noTM

S2 Fig. Analysis of the deduced amino acid sequence of *PSPTO_4977* gene with SignalP 4.1 Server (DTU Bioinformatics). SignalP 4.1 predicted the presence of a signal peptide at the amino terminus of PSPTO_4977. The potential peptide cleavage site is located between amino acids 21 and 22 with a discrimination score (D-score) of 0.884, well-above the threshold score (D-cutoff) of 0.570.
