# Supplementary material for: The Pseudomonas syringae pv. tomato DC3000 PSPTO_0820 multidrug transporter is involved in resistance to plant antimicrobials and bacterial survival during tomato plant infection
Source: PLoS One. 2019 Jun 25;14(6):e0218815. doi: 10.1371/journal.pone.0218815 (PMC6592562; doi:10.1371/journal.pone.0218815)
Supplement: S3 Fig — (PDF) [file pone.0218815.s007.pdf]

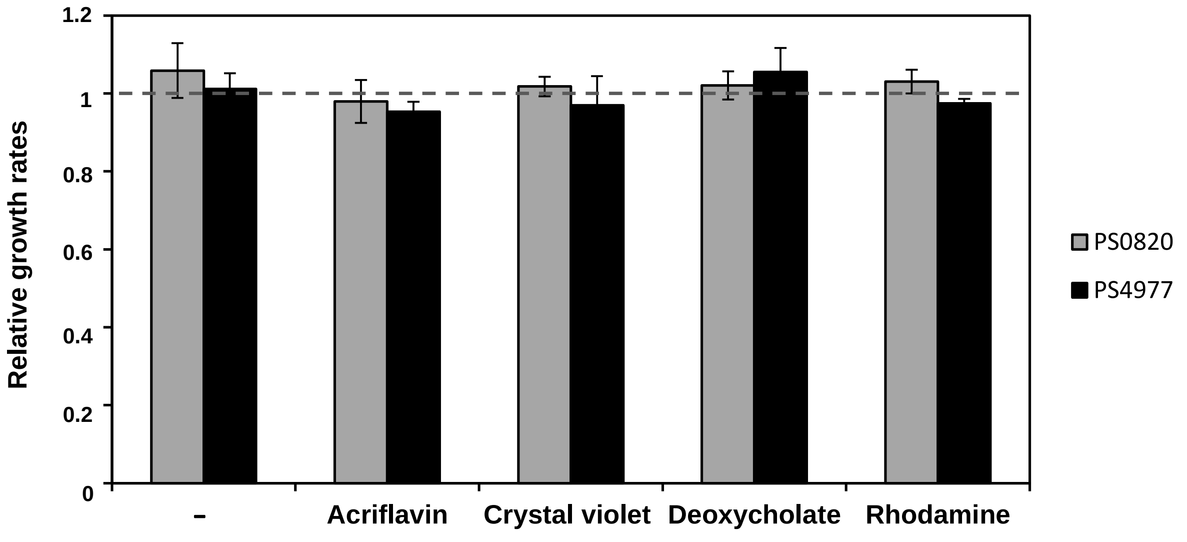

S3 Fig. Effect of antimicrobial compounds on MDR mutant growth as compared to the *PsPto* wild-type. Bacterial growth was determined after 24 h of incubation in LB medium at 28°C by measuring the culture optical density at 600 nm (OD_{600}). The relative growth ratios of the mutants were compared to that of the wild-type, which was set to 1. Data represent the means and standard errors of at least three independent replicates. No significant differences were observed between the wild-type strain and the MDR mutant strains.
