# Supplementary material for: The Pseudomonas syringae pv. tomato DC3000 PSPTO_0820 multidrug transporter is involved in resistance to plant antimicrobials and bacterial survival during tomato plant infection
Source: PLoS One. 2019 Jun 25;14(6):e0218815. doi: 10.1371/journal.pone.0218815 (PMC6592562; doi:10.1371/journal.pone.0218815)
Supplement: S4 Fig — (PDF) [file pone.0218815.s008.pdf]

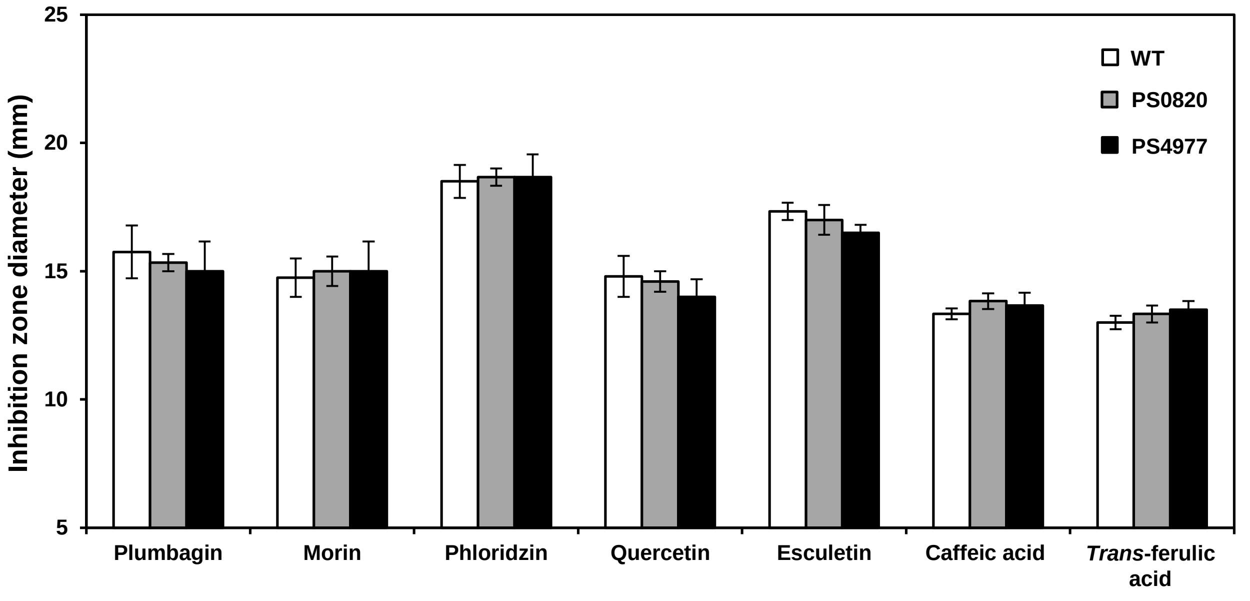

S4 Fig. Susceptibility of *PsPto* wild-type and MDR mutants to other alkaloids and phenylpropanoids. Bacterial cells were inoculated onto LB plates in the presence of different plant antimicrobial solutions deposited into a well at the center of the plate. The diameter of the growth inhibition halo was measured after 24 h incubation at 28°C. Data represent the means and standard errors of at least three independent replicates. No significant differences were observed between the wild-type strain and the MDR mutant strains. For berberine, rhein, genistein, naringenin and phloretin not growth inhibition was observed at the maximum concentrations tested.
