# Supplementary material for: The Pseudomonas syringae pv. tomato DC3000 PSPTO_0820 multidrug transporter is involved in resistance to plant antimicrobials and bacterial survival during tomato plant infection
Source: PLoS One. 2019 Jun 25;14(6):e0218815. doi: 10.1371/journal.pone.0218815 (PMC6592562; doi:10.1371/journal.pone.0218815)
Supplement: S5 Fig — (PDF) [file pone.0218815.s009.pdf]

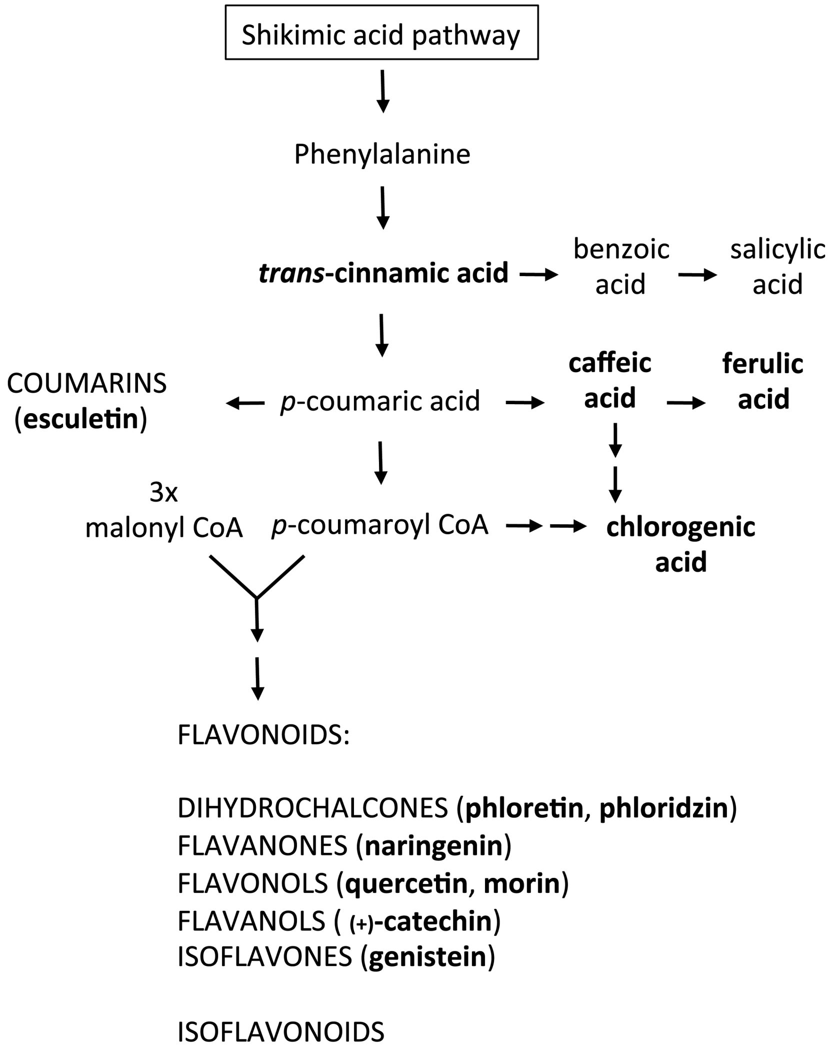

S5 Fig. Schematic view of the main biosynthetic pathways leading to phenylpropanoid products in plants. Enzyme names have been omitted for simplicity. Phenylpropanoids derive from the amino acid L-phenylalanine via deamination by L-phenylalanine ammonia lyase (PAL). The simplest ones, containing a C_6-C_3 skeleton, are the hydroxycinnamic acids, such as coumaric, caffeic and ferulic acids. The initial three steps of the pathway (from Phe to *p*-coumaroyl CoA) are mandatory and provide the basis for the synthesis of more complex phenylpropanoids, such as flavonoids (C_6-C_3-C_6 skeleton), which are formed by condensation of one coumaroyl- and three malonyl-CoA molecules. In fact, *p*-coumaroyl CoA is one of the most important branchpoints of the route (Vogt T. 2010. Phenylpropanoid biosynthesis. Mol Plant 3:2–20). Names in bold type within the figure indicate some of the phenylpropanoid compounds used in this work.
